# Interleukin 6-regulated macrophage polarization controls atherosclerosis-associated vascular intimal hyperplasia

**DOI:** 10.3389/fimmu.2022.952164

**Published:** 2022-07-27

**Authors:** Jiaquan Chen, Weilun Wang, Qihong Ni, Lan Zhang, Xiangjiang Guo

**Affiliations:** Department of Vascular Surgery, Ren Ji Hospital, Shanghai Jiao Tong University School of Medicine, Shanghai, China

**Keywords:** atherosclerosis, vascular intimal hyperplasia, macrophage polarization, interleukin 6, plaques

## Abstract

Vascular intimal hyperplasia (VIH) is an important stage of atherosclerosis (AS), in which macrophages not only play a critical role in local inflammation, but also transform into foam cells to participate into plaque formation, where they appear to be heterogeneous. Recently, it was shown that CD11c+ macrophages were more associated with active plaque progression. However, the molecular regulation of phenotypic changes of plaque macrophages during VIH has not been clarified and thus addressed in the current study. Since CD11c- cells were M2a-polarized anti-inflammatory macrophages, while CD11c+ cells were M1/M2b-polarized pro-inflammatory macrophages, we used bioinformatics tools to analyze the CD11c+ versus CD11c- plaque macrophages, aiming to detect the differential genes associated with M1/M2 macrophage polarization. We obtained 122 differential genes that were significantly altered in CD11c+ versus CD11c- plaque macrophages, regardless of CD11b expression. Next, hub genes were predicted in these 122 genes, from which we detected 3 candidates, interleukin 6 (Il6), Decorin (Dcn) and Tissue inhibitor matrix metalloproteinase 1 (Timp1). The effects of these 3 genes on CD11c expression as well as on the macrophage polarization were assessed *in vitro*, showing that only expression of Il6, but not expression of Dcn or Timp1, induced M1/M2b-like polarization in M2a macrophages. Moreover, only suppression of Il6, but not suppression of either of Dcn or Timp1, induced M2a-like polarization in M1/M2b macrophages. Furthermore, pharmaceutical suppression of Il6 attenuated VIH formation and progression of AS in a mouse model that co-applied apolipoprotein E-knockout and high-fat diet. Together, our data suggest that formation of VIH can be controlled through modulating macrophage polarization, as a promising therapeutic approach for prevent AS.

## Introduction

Atherosclerosis (AS) is a leading cause for heart attack, ischemic stroke and lower extremity arterial sclerosis obstruction disease (1). During the development of AS, macrophages not only play a critical role in the local inflammation, but also transform into foam cells to participate into the plaque formation (2). Vascular Intimal hyperplasia (VIH) is a process in which the vascular intima becomes thickened due to the appearance of unexpected cells and proteoglycan-rich extracellular matrix between the internal elastic lamina of the vessels and vascular endothelia (3). It has been found that VIH increases lipoprotein retention in the vascular intima and accelerated atherosclerosis, and thus becomes an initial pathological process for AS development (4).

Injuries on the vascular wall by hypertension, aging and local and systemic inflammation have all been regarded as important factors to boost formation of VIH (5). Since inflammation is a well-known contributor to VIH, macrophages, as one of the major players in both acute and chronic inflammation, are supposed to play crucial roles during VIH (6). However, the function and regulation of macrophages, in terms of the control of their differentiated status (polarization), are not fully determined (2).

VIH is likely the early stage of plaques. VIH contains cell types including macrophages that could transform into foam cells, which are critical for plaque formation (7). It is known that plaque macrophages are heterogeneous (4). Recently, it was shown that CD11c+ macrophages appeared to be more involved in active plaque progression (8). Moreover, this study also demonstrated that CD11c- cells were M2a-polarized anti-inflammatory macrophages, while CD11c+ cells were M1 and M2b-polarized pro-inflammatory macrophages (8). However, the molecular regulation of phenotypic changes of plaque macrophages during VIH has not been clarified and thus addressed in the current study.

## Materials and methods

### Ethics

This study was approved by the Animal Care and Use Committee of Shanghai Jiao Tong University. All experimental procedures were performed in accordance with the Guide for the Care and Use of Laboratory Animals, published by the US National Institutes of Health.

### Animal models

Apolipoprotein E (ApoE)-knockout mice were purchased from Laboratory Animal Center of Peking University Health Science Center (Beijing, China). Twelve-week-old male and female mice were used in the experiments. The animals were randomly grouped as following: normal diet (ND), high-fat diet (HFD, including 21% fat and 0.25% cholesterol), HFD with intraperitoneal injection of control IgG every other day (HFD+IgG), and HFD with intraperitoneal injection of control Siltuximab (10mg/kg, SYLVANT, US) every other day (HFD+ Siltuximab). Each group contained 3 males and 3 females. All animals were maintained for 3 months before analysis.

### Atherosclerotic occurrence and severity

The mouse femoral artery was excised under the microscope, after which it was fixed with 4% paraformaldehyde for 6 hours before paraffin processing and sectioning for 4μm-thickness slides. AS lesions were examined by Oil red O staining (Oil red O staining kit, Invitrogen, CA, Carlsbad, USA) followed by hematoxylin counterstaining. The data were obtained from all 6 mice (3 males and 3 females) from each experimental group.

### *In vitro* culture, differentiation and transfection of macrophages

Mouse macrophages were isolated from bone marrow from 12-week-old male ApoE-knockout mice. The bone marrow was flushed out by a 5ml syringe using Iscove’s Modified Dulbecco’s Media (IMDM) containing 8% heat inactivated fetal bovine serum (FBS) into culture with same media containing 10 ng/mL of macrophage colony stimulating factor (M-CSF, Invitrogen) for seven days. M1/M2b differentiation of mouse macrophages were induced by addition of interferon (IFN)-γ (50 ng/ml, Invitrogen), lipopolysaccharides (LPS, 10 ng/ml, Invitrogen) and interleukin (Il)1β (10 ng/ml, Invitrogen). M2a differentiation of mouse macrophages were induced by addition of 20 ng/ml Il4 (Invitrogen). Plasmids expressing Il6, Decorin (Dcn) and Tissue inhibitor matrix metalloproteinase 1 (Timp1) and their siRNAs were generated with their cDNA as templates and corresponding sequences, respectively. Transfection of macrophages was done for 48 hours with Lipofectamine 2000 (Invitrogen).

### Flow cytometry

The dissected VIH/plaques from mouse femoral arteries were dissociated with 0.25% Trypsin to obtain a single cell fraction to be double stained with FITC-conjugated F4/80 and PE-cy7-conjugated CD11c antibodies (Becton-Dickinson Biosciences, San Jose, CA, USA) for 15 minutes before analysis and sorting with a FACScan flow cytometer (Becton-Dickinson Biosciences).

### RT-qPCR

Total RNA was isolated with RNeasy mini kit (Qiagen, Beijing, China). The cDNA was generated using isolated RNAs as templates (β-actin as housekeeping gene), and then subjected to real-time quantitative PCR (RT-qPCR) with a QuantiTect SYBR Green PCR Kit (Qiagen). All primers were Qiagen commercial ones. Relative expression levels were shown.

### Statistical and bioinformatic analyses

Differences between two groups were compared using student’s T test with a GraphPad Prism software (GraphPad Software, Inc. La Jolla, CA, USA) and shown as the individual values in the figures. p < 0.05 was considered significant. No significance was shown as n.s. Bioinformatics on GEO database (number GEO137819) were performed using a combination of R language, a Metascape online pathway tool, an online tool for Veen analysis from Bioinformatics & Evolutionary Genomics, a String online protein interaction analytic tool and Cytoscape online tools.

## Result

### Using bioinformatic tools to analyze different subtypes of plaque macrophages

**Figure 1:**
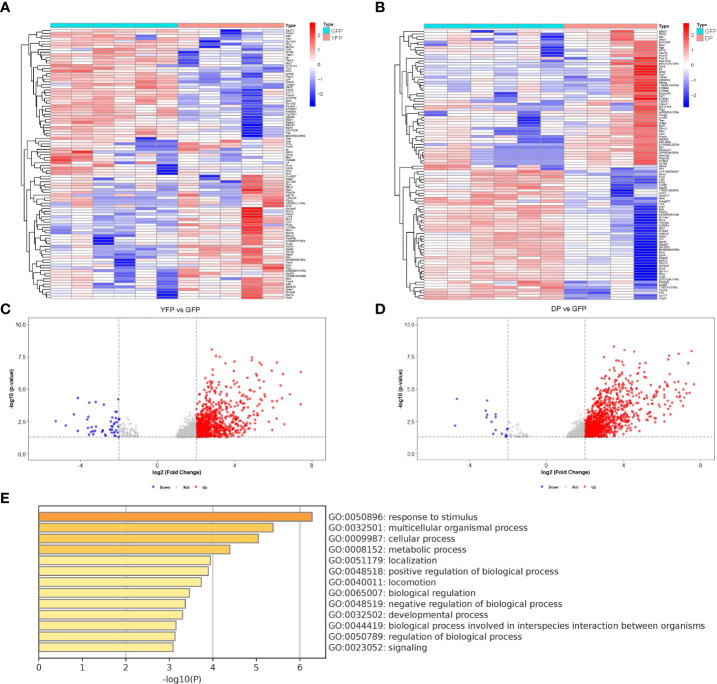
Using bioinformatic tools to analyze different subtypes of plaque macrophages. We analyzed the online database (GEO137819). In this study, combined transgenic mice to label CD11c yellow fluorescent protein (YFP) and to label CD11b green fluorescent protein (GFP)) were used to separate plaque macrophages into 4 populations, CD11c+CD11b+ (double positive for YFP and GFP, DP) macrophages, CD11c+CD11b- (YFP) macrophages, CD11c-CD11b+ (GFP) macrophages and CD11c-CD11b- (double negative for YFP and GFP, DN) macrophages. **(A, B)** Heat map showed of diff genes in YFP cells versus GFP cells **(A)** and diff genes in DP cells versus GFP cells **(B)**. **(C, D)** Volcano map showed of diff genes in YFP cells versus GFP cells **(C)** and diff genes in DP cells versus GFP cells **(D)**. **(E)** The most affected pathways by analysis of the shared diff genes from YFP cells versus GFP cells and from DP cells versus GFP cells (568 in total) with Metascape online tools.

Plaque macrophages are heterogeneous and only recently, CD11c+ macrophages were found to be more involved in active plaque progression, compared to CD11c- macrophages (8). In this study, genetic tools [combined transgenic mice to label CD11c yellow fluorescent protein (YFP) and to label CD11b green fluorescent protein (GFP)] were used to separate plaque macrophages into 4 populations, CD11c+CD11b+ (double positive for YFP and GFP, DP) macrophages, CD11c+CD11b- (YFP) macrophages, CD11c-CD11b+ (GFP) macrophages and CD11c-CD11b- (double negative for YFP and GFP, DN) macrophages (8). GFP macrophages were likely M2a-polarized anti-inflammatory macrophages, while YFP macrophages were likely M1/M2b-polarized pro-inflammatory macrophages (8). While GFP cells appeared to be associated with quiescent plaques, YFP and DP cells appeared to be associated with activated plaques (8). However, the molecular regulation of phenotypic changes of these plaque macrophages was not addressed in that study (8). To address this question, we studied the online database (GEO137819) from this study. We did two comparisons, first YFP cells versus GFP cells, and second, DP cells versus GFP cells. The purpose of these comparisons was to figure out the significantly altered genes (differential genes or diff genes) in macrophages that may be associated with the activation of the plaques. The diff genes from two comparisons were obtained by R languages and then shown in heat maps (**Figures 1A, B**), and in volcano maps (**Figures 1C, D**). The shared diff genes (568 in total) from the two comparisons were used to analyze for the most affected pathways with Metascape online tools (**Figure 1E**). Of note, many of highly altered pathways contained diff genes associated with macrophage polarization.

### Using bioinformatic tools to identify candidate genes for controlling plaque macrophage polarization

**Figure 2:**
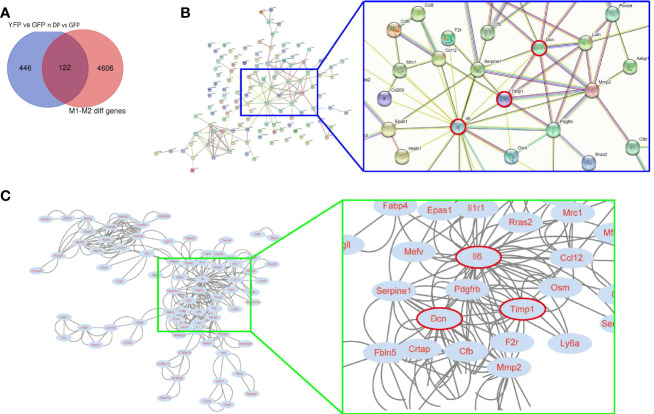
Using bioinformatic tools to identify candidate genes for controlling plaque macrophage polarization. **(A)** An online tool from Bioinformatics & Evolutionary Genomics for Veen analysis was applied to find 122 genes from the 568 diff genes associated with the control of macrophage polarization. **(B, C)** The 122 genes were then put into a protein interaction analysis using a String online protein interaction analytic tool, showing that Il6, Dcn and Timp1 were 3 nodule proteins in the protein interaction network. A blue rectangle region was enlarged to visualize the position of Il6, Dcn and Timp1 (red circled) in the network. **(C)** A Cytoscape online tool was used to confirm that Il6, Dcn and Timp1 were hub proteins that may be important for the protein interaction among these diff genes. A green rectangle region was enlarged to visualize the position of Il6, Dcn and Timp1 (red circled) in the network.

Next, we tried to identify the diff genes that regulate macrophage polarization. We used an online tool from Bioinformatics & Evolutionary Genomics for Veen analysis and found that from the 568 diff genes, there were 122 genes associated with the control of macrophage polarization (**Figure 2A**). These 122 genes were then put into a protein interaction analysis using a String online protein interaction analytic tool, showing that Il6, Dcn and Timp1 were 3 nodule proteins in the protein interaction network (**Figure 2B**). Further analysis with Cytoscape online tools further confirmed that these 3 proteins were hub proteins that may be important for the protein interaction among these diff genes (**Figure 2C**). Moreover, these 3 genes were upregulated in YFP and DP cells, compared to GFP cells, and thus may be associated with M1/M2b polarization of macrophages in activating VIH plaques.

### Expression of Il6 induced M1/M2b-like polarization in M2a macrophages

**Figure 3:**
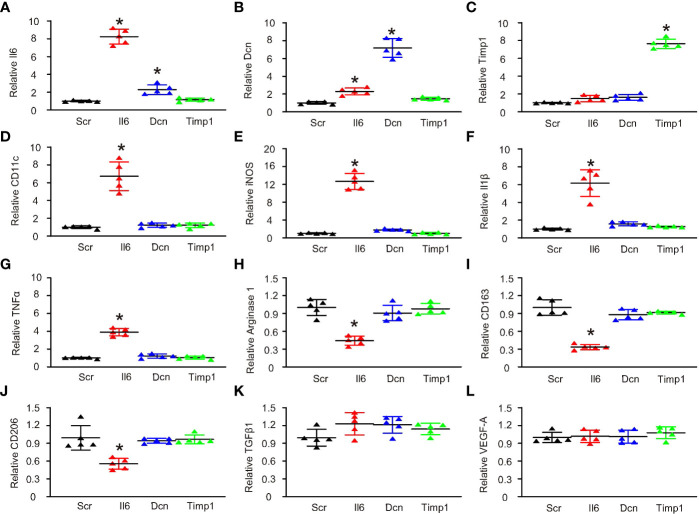
Expression of Il6 induced M1/M2b-like polarization in M2a macrophages. Overexpressing plasmids for Il6, Dcn and Timp1 and a control scramble plasmid (Scr) were used to treat M2a macrophages, which were induced by Il4. Gene expression was examined by RT-qPCR. **(A–L)** RT-qPCR for Il6 **(A)**, Dcn **(B)**, Timp1 **(C)**, CD11c **(D)**, iNOS **(E)**, Il1β **(F)**, TNFα **(G)**, arginase 1 **(H)**, CD163 **(I)**, CD206 **(J)**, TGFβ1 **(K)** and VEGF-A **(L)**. *p < 0.05.

In order to validate the importance of these 3 genes in the control of CD11c expression as well as in the alteration of macrophage polarization, we used their overexpressing plasmids to treat M2a macrophages, which were induced by Il4. First, overexpressing plasmids were validated by expression of these 3 genes by transfection of M2a macrophages (**Figures 3A-C**). Interestingly, Il6 and Dcn seemed to induce expression of each other significantly but weakly (**Figures 3A, B**), while Timp1 did not change expression of Il6 and Dcn (**Figure 3C**). Next, we found that only Il6 significantly increased the levels of CD11c (**Figure 3D**). Moreover, only Il6 significantly increased the levels of iNOS (**Figure 3E**), Il1β (**Figure 3F**) and TNFα (**Figure 3G**), which were 3 marker genes for M1/M2b macrophage polarization. In addition, only Il6 significantly decreased the levels of arginase 1 (**Figure 3H**), CD163 (**Figure 3I**) and CD206 (**Figure 3J**), which were 3 marker genes for M2a macrophage polarization. Furthermore, transforming growth factor β1 (TGFβ1) (**Figure 3K**) and vascular endothelial growth factor A (VEGF-A) (**Figure 3L**), two key genes for M2c and M2d polarization respectively, were unchanged. Thus, expression of Il6, but not expression of Dcn or Timp1, induced M1/M2b-like polarization in M2a macrophages.

### Suppression of Il6 induced M2a-like polarization in M1/M2b macrophages

**Figure 4:**
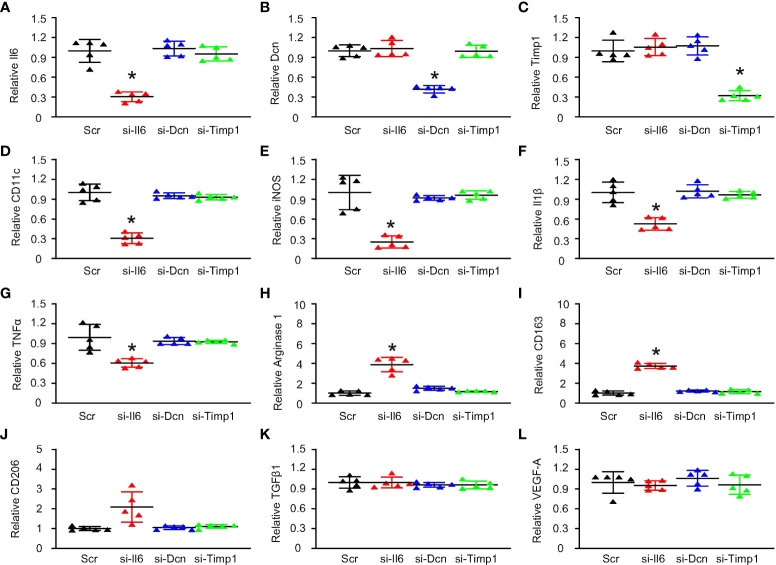
Suppression of Il6 induced M2a-like polarization in M1/M2b macrophages. SiRNA plasmids for Il6, Dcn and Timp1 and a control scramble plasmid (Scr) were sued to treat M1/M2b macrophages, which were induced by a combination of IFN-γ, LPS and Il1β. Gene expression was examined by RT-qPCR. **(A–L)** RT-qPCR for Il6 **(A)**, Dcn **(B)**, Timp1 **(C)**, CD11c **(D)**, iNOS **(E)**, Il1β **(F)**, TNFα **(G)**, arginase 1 **(H)**, CD163 **(I)**, CD206 **(J)**, TGFβ1 **(K)** and VEGF-A **(L)**. *p < 0.05.

Nex, we used the siRNA plasmids of the 3 genes to treat M1/M2b macrophages, which were induced by a combination of IFN-γ, LPS and Il1β. First, siRNA plasmids were validated by examining the expression of these 3 genes after transfection with corresponding siRNAs (**Figures 4A-C**). Then, we found that only si-Il6 significantly decreased the levels of CD11c (**Figure 4D**). Moreover, only si-Il6 significantly decreased the levels of iNOS (**Figure 4E**), Il1β (**Figure 4F**) and TNFα (**Figure 4G**), which were 3 marker genes for M1/M2b macrophage polarization. In addition, only si-Il6 significantly increased the levels of arginase 1 (**Figure 4H**), CD163 (**Figure 4I**) and CD206 (**Figure 4J**), which were 3 marker genes for M2a macrophage polarization. Furthermore, TGFβ1 (**Figure 4K**) and VEGF-A (**Figure 4L**), two key genes for M2c and M2d polarization respectively, were unchanged. Thus, suppression of Il6, but not suppression of either Dcn or Timp1, induced M2a-like polarization in M1/M2b macrophages. Hence, Il6 appeared to be the key gene that is associated with CD11c expression and macrophage polarization in VIH-associated activating plaques.

### Pharmaceutical suppression of Il6 attenuates VIH formation and progression of AS in a combined ApoE-knockout and HFD mouse model

**Figure 5:**
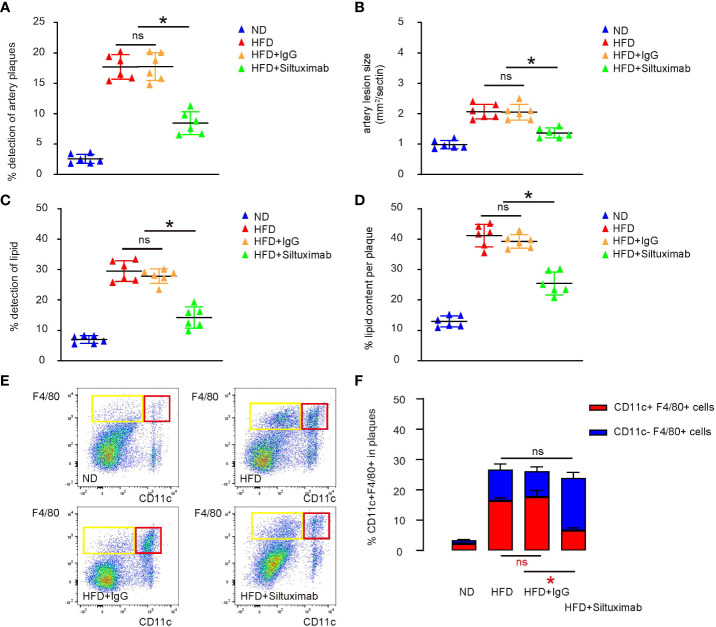
Pharmaceutical suppression of Il6 attenuates VIH formation and progression of AS in a combined ApoE-knockout and HFD mouse model. ApoE-knockout mice were treated with either high-fat diet (HFD) or normal diet (ND) for 12 weeks. Some HFD-treated mice receive either Siltuximab (HFD+ Siltuximab), an FDA-approved monoclonal antibody against both human and mouse Il6, or control IgG (HFD+IgG). The four groups of mice were compared at 3 months. **(A)** Incidence of artery plaque formation. **(B)** Average artery lesion size. **(C)** Incidence of detection of lipid in the vascular intima. **(D)** The plaque lipid content. **(E, F)** Analysis of CD11c in F4/80+ macrophages isolated from the vascular intima, shown by representative flow charts **(E)** and by quantification **(F)**. *p < 0.05. ns, no significance.

ApoE is a potent suppressor of AS, while ApoE-knockout mice display elevated inflammatory responses to long term diet-induced hypercholesterolemia (9). A HFD as long as 3 months has been shown to significantly speed up AS development in ApoE-knockout mice (9). Thus, ApoE-knockout mice were treated with either HFD or ND for 12 weeks. Some HFD-treated mice receive either Siltuximab, an FDA-approved monoclonal antibody against both human and mouse Il6, or control IgG. The four groups of mice were compared at 3 months. We found that the incidence of artery plaque formation (**Figure 5A**), average artery lesion size (**Figure 5B**), the incidence of detection of lipid in the vascular intima (**Figure 5C**) and the plaque lipid content (**Figure 5D**) were all significantly attenuated by Siltuximab administration, compared to control IgG. Macrophages were isolated from the vascular intima, showing that Il6 suppression by Siltuximab significantly reduced CD11c+ macrophages, without changing total macrophage number (**Figures 5E, F**). Together, our data suggest that formation of VIH can be controlled through modulating macrophage polarization, as a promising therapeutic approach for prevent atherosclerosis.

## Discussion

VIH is thought to be the initial pathological event predisposed to development of AS, while cell hyperplasia is likely the early stage of formation of a plaque (1). Since macrophages play a critical role in the development of AS by coordinating local inflammation and by transforming themselves into foam cells (2), it is not surprising that macrophages are also crucial for the process of VIH. Therefore, interfering with VIH and its associated microenvironment may be more effective than treating it at the late stage when AS has already developed.

In the previous study, McArdle et al. nicely showed that plaque macrophages could be separated into CD11c+ versus CD11c- populations (8). Regardless their expression of CD11b, CD11c- macrophages were more likely to be associated with a phenotype close to M2a and were not supporting an acute activating status of plaque formation (8). On the other hand, CD11c+ macrophages were more likely to be associated with a phenotype close to M1 and M2b and were supporting an acute activating status of plaque formation (8). Of note, based on the gene expression profile, it was hard to delicately distinguish M1 polarization from M2b polarization, since macrophages in the plaques are heterogenous and their polarization was spectral rather than uniform (10). Therefore, our finding showing Il6 as a trigger to activate CD11c and the related phenotype may be simply explained as a proinflammatory effect, while our finding showing Il6 suppression to deactivate CD11c and the related M2a-like phenotype may be simply explained as an anti-inflammatory effect (11). The findings on CD11c here are consistent with some previous studies, showing that CD11c was likely expressed in some M1-like macrophages (12, 13) in addition to their traditionally believed expression on dendritic cells (14, 15).

Il6 has been known to play a pivotal role in the early stage of atherosclerotic plaques (16). Il6 could be synthesized and released by many cell types either resident or trapped in the artery wall, e.g. macrophages, vascular smooth muscle and endothelial cells (17). Our *in vivo* results showing that blocking Il6 in the AS-prone mice alleviated VIH and AS progression could be resulted from the inhibition of Il6 in either cell type or from a combined effect. In the future, the importance of Il6 from these cell types could be further investigated using cell-specific promotor-driven suppression of Il6 rather than a pharmaceutical intervention. However, here, our *in vitro* data showed that altering Il6 levels in macrophages were sufficient to modulate the CD11c expression and the phenotypic changes in plaque macrophages. Therefore, regardless of the source of Il6, it plays an important role in the phenotypic determination of plaque macrophage and makes macrophages correspondingly polarized and shaped, ready for contributing to the development of VIH and AS.

## Data Availability Statement

The original contributions presented in the study are included in the article/supplementary material. Further inquiries can be directed to the corresponding author.

## Ethics Statement

The animal study was reviewed and approved by Shanghai Jiao Tong University.

## Author Contributions

XG is responsible for study conception and design, and bioinformatics analysis. All authors are responsible for data acquisition and analysis. XG wrote the manuscript and all authors have read the manuscript and agreed with the publication. XG is responsible for funding and are the guarantee of the study.

## Conflict of Interest

The authors declare that the research was conducted in the absence of any commercial or financial relationships that could be construed as a potential conflict of interest.

## Publisher’s Note

All claims expressed in this article are solely those of the authors and do not necessarily represent those of their affiliated organizations, or those of the publisher, the editors and the reviewers. Any product that may be evaluated in this article, or claim that may be made by its manufacturer, is not guaranteed or endorsed by the publisher.
